# Chikungunya nsP2 protease is not a papain-like cysteine protease and the catalytic dyad cysteine is interchangeable with a proximal serine

**DOI:** 10.1038/srep17125

**Published:** 2015-11-24

**Authors:** Chonticha Saisawang, Sawanan Saitornuang, Pornpan Sillapee, Sukathida Ubol, Duncan R. Smith, Albert J. Ketterman

**Affiliations:** 1 Institute of Molecular Biosciences, Mahidol University, Salaya Campus, Thailand; 2 Department of Microbiology, Faculty of Science, Mahidol University, Bangkok, Thailand; 3 Center for Emerging and Neglected Infectious Diseases, Mahidol University, Thailand

## Abstract

Chikungunya virus is the pathogenic alphavirus that causes chikungunya fever in humans. In the last decade millions of cases have been reported around the world from Africa to Asia to the Americas. The alphavirus nsP2 protein is multifunctional and is considered to be pivotal to viral replication, as the nsP2 protease activity is critical for proteolytic processing of the viral polyprotein during replication. Classically the alphavirus nsP2 protease is thought to be papain-like with the enzyme reaction proceeding through a cysteine/histidine catalytic dyad. We performed structure-function studies on the chikungunya nsP2 protease and show that the enzyme is not papain-like. Characterization of the catalytic dyad cysteine residue enabled us to identify a nearby serine that is catalytically interchangeable with the dyad cysteine residue. The enzyme retains activity upon alanine replacement of either residue but a replacement of both cysteine and serine residues results in no detectable activity. Protein dynamics appears to allow the use of either the cysteine or the serine residue in catalysis. This switchable dyad residue has not been previously reported for alphavirus nsP2 proteases and would have a major impact on the nsP2 protease as an anti-viral target.

Chikungunya virus (CHIKV) is mosquito-borne, and a major pathogenic agent that causes chikungunya fever in humans. CHIKV originated in Africa and spread rapidly into Asia and Europe. Within the last 10 years, a number of Chikungunya outbreaks have arisen in various parts of the world^1^. Recently, more than a million suspected and confirmed cases have been reported in the Americas^2^. Although many of the reported cases were from travelers who brought back the disease from the affected countries where they had visited, autochthonous infections were also reported^2^,^3^,^4^. This alphavirus is primarily transmitted by *Aedes aegypti* and *Aedes albopictus* mosquito vectors and humans are an amplifying host^5^. Patients infected with CHIKV will develop symptoms some 3–5 days after infection. Common symptoms are characterized by joint and muscle pain, rash, nausea and headache. The fever and pain may persist for months and occasional fatalities have been reported. The CHIKV genome is comprised of a positive sense single-stranded RNA which contains two open reading frames encoding the non-structural and structural proteins^6^. The non-structural polyprotein precursor, nsP123 or nsP1234, is translated first and is proteolytically cleaved into 4 individual mature proteins by the virus-encoded nsP2 protease in a specific manner while the structural proteins are subsequently translated from a subgenomic mRNA^7^.

The alphavirus nsP2 protein is a multifunctional enzyme with the N-terminus of the protein comprising of RNA helicase, nucleoside triphosphatase (NTPase) and RNA-dependent 5′-triphosphatase activities^8^,^9^,^10^; whereas, the C-terminus of nsP2 contains the protease domain^11^,^12^. nsP2 is considered to be an essential protein as it is responsible for viral replication and propagation with its proteolytic and other activities^13^. In addition nsP2 is translocated to the nucleus where it shuts down antiviral gene expression^14^,^15^. The proteolytic activity of nsP2 has been characterized in other alphaviruses, such as Sindbis virus (SINV), Semliki forest virus (SFV) and Venezuelan Equine Encephalitis virus (VEEV), as a papain-like cysteine protease with a cysteine-histidine catalytic dyad in the active site^11^,^12^,^16^,^17^,^18^,^19^. This led to the suggestion that the cysteine-histidine catalytic dyad possesses a nucleophilic cysteine residue that catalyzes the peptide bond cleavage with a histidine residue serving as a general base in the reaction. Cysteine proteases or thiol proteases are comprised of different families each of which cluster into clans based on sequence identities, similarities and 3D-structure^20^. The alphavirus nsP2 protease is classified into the Togavirus cysteine endopeptidase family (C9) which belongs to clan CN^21^.

To date there is no vaccine against CHIKV and as the multifunctional nsP2 protein is critical for viral replication it makes an attractive potential anti-CHIKV drug target. To this end we have begun characterization of the protease active site with the initial focus on the catalytic dyad. Although the dyad residues have been identified previously in other alphavirus nsP2 proteins; in SINV, SFV and VEEV^11^,^12^,^16^,^17^,^18^, the CHIKV nsP2 protease active site has not been experimentally characterized. In performing this study a structural comparison showed that CHIKV nsP2 protease is not papain-like, and we have found what appears to be a unique feature of CHIKV nsP2 protease, in which the cysteine dyad residue can be catalytically replaced by a vicinal serine.

## Results and Discussion

**Figure 1:**
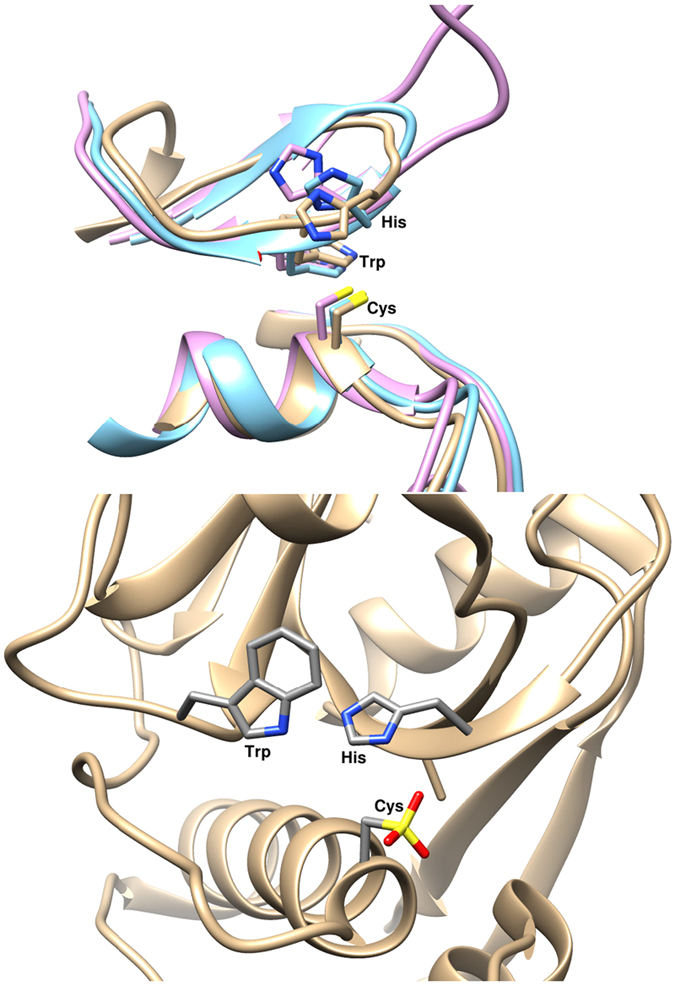
Active sites of 3 alphavirus nsP2 proteases and papain. The upper panel shows superposition of the 3 currently available alphavirus nsP2 protease active sites. Tan color ribbon is CHIKV nsP2 (PDB ID: 3TRK), blue ribbon is VEEV nsP2 (PDB ID: 2HWK) and purple ribbon is SINV nsP2 (PDB ID: 4GUA). The lower panel shows the active site of papain (PDB ID: 9PAP). Molecular graphics and analyses were performed with the UCSF Chimera package. Chimera is developed by the Resource for Biocomputing, Visualization, and Informatics at the University of California, San Francisco (supported by NIGMS P41-GM103311)^49^.

Previously the alphavirus nsP2 protease enzyme has been defined as a papain-like cysteine protease^16^,^22^,^23^,^24^,^25^. As it appeared that the nsP2 was a cysteine-histidine dyad protease similar to papain, the papain protease has been used as a model for which structure was available for several decades. Currently, there are 3 available alphavirus nsP2 protease structures, for Venezuelan equine encephalitis virus (VEEV; PDB ID: 2HWK), Sindbis virus (SINV; PDB ID: 4GUA) and Chikungunya virus (CHIKV; PDB ID: 3TRK). Structural superposition with papain and these nsP2 structures cannot be performed beyond 4 atom pairs which supports that these alphavirus protein motifs are not related to papain (Fig. 1). However, structural superposition of CHIKV nsP2 protease domain to the VEEV protease (245 atom pairs) gives an RMSD of 1.122 Å and to SINV protease (250 atom pairs) an RMSD of 1.024 Å. The superposition of the three alphavirus nsP2 proteases demonstrates a highly conserved tertiary structure for the nsP2 proteases despite the amino acid sequence variations, for example, CHIKV nsP2 pro aligned with SINV and VEEV nsP2 pro shows 44% and 42% amino acid identity, respectively. Of interest in this report is the sequence difference as this is what makes the CHIKV nsP2 different from the other studied alphavirus nsP2 proteases.

Several studies have been reported characterizing the SFV, SINV, VEEV and CHIKV truncated nsP2 protease domain alone^8^,^24^,^26^,^27^,^28^. Moreover, the SFV full length nsP2 and protease domain have been stated to be similar^29^. It was reported for SFV nsP2 that “The high solubility and specific activity of Pro39 suggest that this fragment represents a structurally compact protease domain of nsP2”^26^. The requirement for the N-terminus of nsP2 has only been demonstrated for SFV and in the same study nsP2 protease from SINV processed the SFV polyprotein but the SFV nsP2 could not process the SINV polyprotein^30^. These reports as well as the amino acid identity differences mentioned above demonstrate fundamental differences in the alphavirus nsP2 proteases. We have previously demonstrated that the full length CHIKV nsP2 and the truncated protease domain are not significantly different for protease activity^31^. In our previous report we demonstrated that the CHIKV nsP2 protease (truncated and full length) could recognize small peptide substrates, which has not been reported for Sindbis virus (SINV) or Semliki forest virus (SFV) nsP2 protease. Moreover, the CHIKV nsP2 protease recognition of small substrates also has been shown previously by another group^8^. Therefore an additional difference noted for CHIKV nsP2 is the length of the substrates that can be cleaved by the protease activity^31^. The substrates employed in the present study are the biological cleavage site sequences of the CHIKV protease with FRET tags; which is unlike previous reports for SFV and SINV nsP2 that use large GFP and thioredoxin fusion tagged sequences^29^,^30^,^31^,^32^,^33^. The SFV nsP2 showed no cleavage of 2/3 sequence unless 170 amino acids of the N-terminus of nsP3 was part of the substrate^33^. Although even the EGFP-170 nsP3 substrate showed incomplete cleavage suggesting the lack of activity for 2/3 cleavage could be due to steric hindrance by the presence of the thioredoxin fusion protein and, in any case, demonstrating specificity/activity differences compared to the other two SFV cleavage sites. In addition, CHIKV nsP2-Pro compared with the same region of SFV and SINV shows only 65% and 44% amino acid identity, respectively^31^. The amino acid identity differences in the nsP2 proteins would by itself logically suggest that the nsP2 proteases will be different. Moreover, the SFV cleavage sequences are actually different from the CHIKV sequences, for example the important SFV P5 residue of the 1/2 site, is Tyr; but, in CHIKV it is Asp. In fact, in the literature no data is presented concerning the testing of small peptides as substrates as all activity shown is with large fusion constructs. Verification of our *in vitro* findings in an *in vivo* context is the logical next study. The current study clearly demonstrates properties that the protease domain possesses which implies that these properties may also be expressed *in vivo* and this is the obvious justification for all recombinant protein characterization studies.

**Figure 2:**
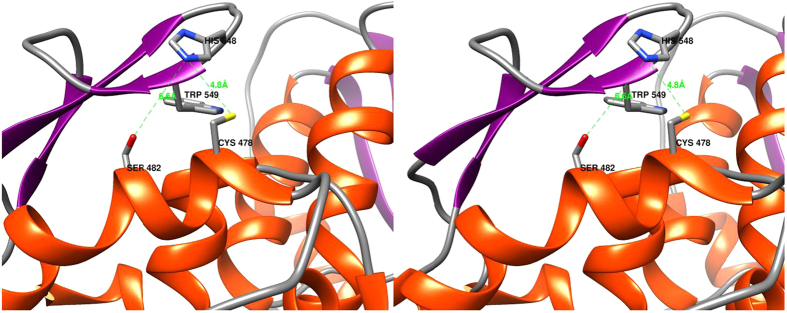
Stereoview the CHIKV nsP2-Pro (PDB ID: 3TRK) active site. Displayed are the canonical Cys/His dyad and the exchangeable Ser. The Trp orientation would preclude direct involvement in the active site. Distances are in Angströms in green. Alpha helices are shown in red and beta strands in purple. Molecular graphics and analyses were performed with the UCSF Chimera package. Chimera is developed by the Resource for Biocomputing, Visualization, and Informatics at the University of California, San Francisco (supported by NIGMS P41-GM103311)^49^.

Several initial studies have shown cysteine and histidine residue involvement with catalysis, which gave nsP2 the papain-like designation^16^,^22^. As papain has been extensively studied (for example^34^,^35^,^36^) and the alphavirus nsP2 has been characterized as papain-like there appear to have been few studies to characterize the kinetic mechanism for the nsP2 protease. This is unfortunate as now available structures and the data in this report demonstrate that alphavirus nsP2 is not papain-like. For example, a tryptophan residue in papain has been shown to make an important contribution to the catalytic mechanism^36^,^37^. In the early literature for alphavirus, a tryptophan residue was also found to cause nsP2 enzyme activity loss, similar to what had been shown for the papain catalytic mechanism^11^. Later, the tryptophan residue was suggested to be involved with nsP2 protease substrate recognition of the glycine residue in the P2 position^24^. Now, the availability of the nsP2 protease structures shows that the tryptophan residue is actually in the wrong orientation to participate in catalysis. The tryptophan residue appears to be in a pocket surrounded by hydrophobic residues and van der Waals contacts (3.3 to 4.1 Å in the CHIKV nsP2, PDB ID: 3TRK); therefore, it is most likely involved in structural stability of the loop that contains the catalytic histidine residue (Figs 1 and 2). To confirm this role we replaced the tryptophan residue with alanine and phenylalanine and performed characterization studies with the two proteins. The kinetic parameters obtained for the 2 tryptophan position mutants show differences for the two engineered proteins for the 3 substrates (Table 1). In contrast to the previous reports for the other alphavirus nsP2 enzymes neither mutant showed a total loss of activity for any substrate. Perhaps surprisingly, the Trp549Ala enzyme actually showed a 3-fold increase in activity for the AGC substrate (Table 1). The alanine or phenylalanine residues affected the kinetic parameters to a different extent, and these effects also appeared to be substrate dependent. For example, both mutants showed decreased activity for AGA but also an increase in affinity as shown by a decreased K_m_ value such that the catalytic efficiency ratio (k_cat_/K_m_) for both enzymes increased compared to the wild type enzyme (Table 1). But both enzymes showed quite different effects on kinetic parameters with the AGC substrate; the Trp549Ala enzyme had increased activity, the Trp549Phe had decreased activity and both had similar K_m_’s compared to wild type enzyme parameters (Table 1). Both tryptophan position mutants, for the AGG substrate, showed similar activity as the wild type but displayed affinity (K_m_) differences with Trp549Phe similar to wild type but Trp549Ala showing decreased affinity with an increased K_m_ (Table 1).

We used several classical protease inhibitors as well as metal salts to characterize the nsP2 enzymes. Although the alphavirus nsP2 has been classified as a cysteine protease there are many studies that show bacterial and viral cysteine proteases behave differently to papain-like cysteine proteases, such that the enzymes show little to no inhibition to E-64, chymostatin, PMSF or leupeptin^38^,^39^,^40^,^41^,^42^,^43^,^44^,^45^. Therefore to further characterize the tryptophan mutants we used several protease inhibitors and metal ions to observe the effects on the activity with the 3 substrates (Tables 2 and 3). Both tryptophan mutants appeared to behave similar to the wild type enzyme in the presence of the protease inhibitors (Table 2). The metal ion effects on the activity of the 2 tryptophan mutants for the 3 substrates suggested more complex conditions existed (Tables 2 and 3). Perhaps cobalt and zinc effects illustrate this complexity best. Cobalt showed different effects depending on the substrate employed, with enhanced activity for AGA, inhibition for AGC and moderate increased activity for AGG (Table 3). The tryptophan residue mutants responded differently to each other as well as compared to the wild type suggesting topological changes in the active site upon protein interaction with the metal. With zinc these interactions allowed the tryptophan residue mutants to retain greater activity for all 3 substrates (Table 3).

The modest effects of the tryptophan residue mutations on the kinetic parameters support the idea that the residue position does not play a direct role in catalysis. We suggest that the tryptophan residue position is involved in protein dynamics through anchoring of the loop that contains the catalytic dyad histidine residue. The residue changes in this position affected the conformational ensembles of the proteins, which indirectly affected the enzyme catalysis and affinity of interaction with the substrates.

Initial experiments to confirm the catalytic dyad cysteine residue were performed with the residue replaced with alanine. Surprisingly the engineered enzyme did not lose all activity as had been reported for SINV and SFV alphavirus nsP2 proteases^11^,^12^. Analysis of the available CHIKV nsP2 protease structure (PDB ID: 3TRK) suggested that a serine residue one helical turn away from the cysteine residue may be substituting in the catalytic role (Fig. 2). In the CHIKV structure this serine is 6.6 Å from the dyad histidine residue (Ser OG to His NE2) which seems too far. However, examination of the VEEV (PDB ID: 2HWK) and SINV (PDB ID: 4GUA) nsP2 protease structures show that the distances between the atoms of the dyad cysteine (SG) and histidine (NE2) residues range from 5.6 to 8.1 Å. This suggests dynamic movement of the static crystal structure conformations must occur to position the catalytic residues correctly and these types of movements could also account for placing the CHIKV serine residue an equivalent distance into a catalytic position. To test the hypothesis of serine catalysis we also generated Ser482Ala mutant enzyme as well as the double mutant Cys478Ala/Ser482Ala. Although the double mutant protein had no detectable activity with any substrate, both single mutation enzymes, Cys478Ala and Ser482Ala, had activity for all three substrates (Table 1). Both enzymes, Cys478Ala and Ser482Ala, had a similar k_cat_ to the wild type enzyme for the AGC and AGG substrates (Table 1); however, both enzymes had significantly less activity than wild type for the AGA substrate (Table 1). The binding affinity (K_m_) for AGC and AGG was similar to wild type for both enzymes but was significantly increased (lower K_m_ value) for the AGA substrate. Use of the AGA substrate appears to allow discrimination of the kinetic parameters for the 3 enzymes, Cys478Ala, Ser482Ala and wild type. This suggests that the conformational dynamics of the 3 proteins are different with the changes in k_cat_ of 3- and 9-fold as well as the changes in K_m_ of 6- and 20-fold also demonstrating that other residues (in addition to the ‘dyad’ residues) are significantly involved in the binding and catalysis.

Previously a similar nsP2 protease from the alphavirus SFV was characterized and shown to be completely resistant to E-64, PMSF and leupeptin as well as exhibiting metal ion effects^24^,^26^. In our study the protease inhibitors leupeptin and E-64 showed little to no effect on wild type, Cys478Ala and Ser482Ala enzyme activities for all 3 substrates (Table 2). Chymostatin showed mild inhibition of the wild type for all 3 substrates; but for Cys478Ala and Ser482Ala enzymes the inhibition appeared to be substrate dependent with even a slight enhancement for Ser482Ala activity with AGA (Table 2). PMSF showed the greatest effect on Cys478Ala activity for AGG but little effect on AGA activity of wild type, Cys478Ala and Ser482Ala enzymes (Table 2). The five metal salts studied gave varying effects depending on the metal, the substrate and the enzyme being considered (Table 3). For example, cobalt significantly enhanced AGA activity for wild type and Cys478Ala enzymes but not Ser482Ala, but inhibited AGC activity of all 3 enzymes to a similar extent and gave a mild increase in AGG activity for all 3 enzymes (Table 3). Using substrate equivalent to our AGG (the cleavage site of nsP3/nsP4) the SFV nsP2 showed approximately 80% inhibition with cobalt, whereas for CHIKV we observed a slight enhancement in activity. Previously the SFV nsP2 was reported to be completely inhibited by copper and zinc^26^ and this was also reported for a CHIKV nsP2 protease^8^, however, we observed complete inhibition only for the Ser482Ala enzyme using the substrate AGA in the presence of zinc (Table 3). As previously observed, we suggest that the differences observed for the CHIKV nsP2 proteases originate with the different substrates used, as the different amino acid lengths around the scissile site as well as total substrate length appear to impact on the properties of the enzyme^31^.

A thermal stability study was performed to determine if the residue changes introduced structural properties that could be detected by variations in protein stability. The stability data showed the wild type enzyme and all the engineered mutants in this study behaved in a similar fashion (Table 4). The similarities of the physical properties of the proteins were also corroborated by similar behaviors during expression and purification of the recombinant proteins. The enzyme differences observed in this report would therefore appear to be due to conformational dynamics and the varying ensembles available to each enzyme.

Of specific interest in this report is that the CHIKV nsP2 protease appears able to interchangeably employ either the canonical dyad cysteine residue or a proximal serine residue for catalysis (Fig. 2). The enzyme retained activity with either a Cys478Ala or a Ser482Ala mutation but lost all detectable activity with the double mutation Cys478Ala/Ser482Ala. The data suggests that rather than an induced fit mechanism the substrate interaction employs conformational selection as either the cysteine or serine residue can be utilized for cleavage of all 3 substrates. Some conformations appear to yield greater activity and thereby display a slight residue preference for activity, such as cysteine residue for the AGA substrate cleavage. The cysteine residue appears to have a structural influence as the Cys478Ala mutant shows large experimental variation in the data. Actually, both cysteine and serine residue positions seem to impact on structure to the extent of influencing changes in binding affinity such as for the AGA substrate. The conformational dynamics also were impacted by the metal ions as demonstrated by specificity changes for wild type versus the cysteine and serine residue mutants for the 3 substrates. Several reports show viral protease enzyme activity is affected by metal ions^8^,^19^,^26^,^41^,^44^,^46^. The mechanism is unclear at this time but may be due to the metal interaction with the protein’s amino acid residues (histidine, cysteine, aspartate, glutamate, tyrosine, tryptophan, methionine, serine, threonine, asparagine, glutamine or the main-chain amino and carbonyl groups^47^) impacting on the enzyme’s conformational dynamics which then could yield an increase or decrease in enzyme activity. In our study, for example, cobalt significantly increases activity for AGA substrate for the wild type and Cys478Ala but not Ser482Ala, while inhibiting all 3 protease activities for AGC substrate. These cobalt effects for AGA substrate also support the concept that the wild type enzyme uses serine or cysteine residues interchangeably for catalysis. However, some of the inhibitor data, such as chymostatin, where the wild type activity is greater than either cysteine or serine mutant suggests that the catalytic mechanism is much more complex. Indeed, taken together the data suggests that the available conformation ensembles are impacted by small molecules such as metal ions or inhibitors, as well as the residues present in the active site. These different ensembles present varying structures for the conformational selection process of the substrate binding which then yield the alterations in activity observed. The alphavirus nsP2 protease kinetic mechanism would seem to be more complex than the early literature has suggested. The CHIKV enzyme appears to be unique with the interchangeable cysteine/serine dyad residue; however, characterization of other alphavirus nsP2’s with a similar serine –cysteine arrangement (for example SFV nsP2) needs to be performed. In addition, *in vivo* macromolecular assembly has also been suggested to play an important role in the functionality of nsP2 during viral replication^30^. Obviously, further characterization studies will be necessary if the nsP2 protease will be an anti-viral target.

## Methods

### Recombinant nsP2 protease characterization

The recombinant nsP2 proteases were constructed, expressed and purified as previously described^31^. The engineered mutants were expressed and purified by the same protocol as the wild type protein. Enzyme characterization was performed as previously described^31^. Briefly, the characterization was performed with 3 synthetic fluorescent substrates corresponding to the 3 cleavage sites of the chikungunya viral non-structural polyprotein. These substrates were designated nsP1/nsP2 (AGA), nsP2/nsP3 (AGC) and nsP3/nsP4 (AGG) with the amino acids spanning P4-P5′ of the scissile site (Table 5). These peptides were tagged with a 2-(N-methylamino)benzoyl (Nma) group at the N-terminus and a 2,4- dinitrophenyl (Dnp) group on an additional lysine residue at the C-terminus and were purchase from Peptides International, Inc. Substrate stock solutions were prepared in dimethyl sulfoxide (DMSO) for AGA and AGG and dimethylformamide (DMF) for AGC. The substrate stock solutions were diluted in assay buffer (50 mM Tris-HCl, pH 7.5) containing the appropriate solvent, DMSO or DMF, to yield a final constant solvent concentration of 0.4%. Then 90 μl of diluted substrate was transferred a well of a black 96-well plate. Addition of 10 μl purified enzyme (1 μM final concentration for AGC and AGG; 2 μM for AGA assay) started the reaction. Protease activity was followed by continuously monitoring for 3 hr increasing fluorescence (substrate depletion) using a Beckman Coulter DTX880 multimode detector at 340 nm excitation and 430 nm emission wavelengths at 37 °C. To obtain the kinetic parameters, control curves with no enzyme were subtracted from the enzyme progress curves, which were then analyzed by Dynafit program^©^ version 3.28.070 (BioKin, Ltd.)^48^.

### Metal and protease inhibitor characterization

The effects on nsP2 protease activity of metal ions and protease inhibitors were characterized as previously described^31^. Briefly, a single concentration of 2 mM metal ion was employed to test cobalt (Co^2+^), magnesium (Mg^2+^), zinc (Zn^2+^), nickel (Ni^2+^) and copper (Cu^2+^) effects on protease activity. Different final concentrations of four protease inhibitors were used; 50 μM chymostatin (inhibits chymotrypsin-like proteases), 10 μM E-64 (a selective cysteine protease inhibitor), 100 μM leupeptin (inhibits serine and thiol proteases) and 1 mM phenylmethanesulfonyl fluoride (PMSF; a commonly used serine protease inhibitor). The enzyme activity in the presence of the metal ions or inhibitors was measured with the 3 fluorescent substrates. The initial reaction rate was analyzed using GraphPad Prism^®^ software, version 5.01. Percent remaining activity was calculated from the enzyme activity obtained in the presence of inhibitor versus the control activity in the absence of inhibitor.

### Stability test

Thermal stability of the nsP2 protease was determined as previously described^31^. Briefly, freshly purified enzyme was incubated at 37 and 42 °C for 10 min then activity was immediately measured using the AGG substrate at 4.5 μM final concentration. The initial reaction rate was evaluated using GraphPad Prism^®^ software, version 5.01. Percent remaining activity was assessed by comparison with the control reaction at 37 °C.

## Additional Information

**How to cite this article**: Saisawang, C. *et al.* Chikungunya nsP2 protease is not a papain-like cysteine protease and the catalytic dyad cysteine is interchangeable with a proximal serine. *Sci. Rep.* **5**, 17125; doi: 10.1038/srep17125 (2015).

## Figures and Tables

**Table 1:** The kinetic parameters for nsP2 pro Wild Type and mutants.

nsP2 pro	k_cat_ (×10^−4^ s^−1^)	K_m_ (μM)	k_cat_/K_m_ (×10^−4^ s^−1^∙μM^−1^)
Substrate AGA			
Wild Type	46.16 ± 8.91	65.73 ± 17.08	0.722 ± 0.151
Cys478Ala	5.150 ± 1.533	3.293 ± 1.048	1.572 ± 0.197
Ser482Ala	16.35 ± 2.91	10.40 ± 2.02	1.637 ± 0.541
Trp549Ala	32.47 ± 7.61	30.14 ± 9.84	1.04 ± 0.35
Trp549Phe	7.59 ± 4.40	4.14 ± 2.02	1.77 ± 0.18
Substrate AGC			
Wild Type	24.21 ± 4.86	6.957 ± 1.607	3.614 ± 1.106
Cys478Ala	31.49 ± 12.71	18.43 ± 16.73	2.417 ± 1.220
Ser482Ala	21.25 ± 11.55	10.92 ± 2.80	1.871 ± 0.577
Trp549Ala	74.89 ± 12.84	7.66 ± 4.56	11.57 ± 5.20
Trp549Phe	12.66 ± 0.53	4.09 ± 1.56	3.36 ± 1.09
Substrate AGG			
Wild Type	16.06 ± 11.45	8.618 ± 5.027	1.792 ± 0.431
Cys478Ala	15.07 ± 7.70	7.172 ± 3.435	2.243 ± 0.854
Ser482Ala	14.33 ± 6.52	5.807 ± 3.646	3.080 ± 1.598
Trp549Ala	16.65 ± 12.39	26.87 ± 9.67	0.67 ± 0.48
Trp549Phe	13.43 ± 1.66	7.59 ± 1.28	1.79 ± 0.24

The kinetic results are represented as mean ± S.D. for at least 3 independent experiments.

**Table 2:** The remaining percent activity of enzyme in the presence of protease inhibitor.

nsP2 pro	AGA	AGC	AGG
Leupeptin			
Wild Type	91.25 ± 2.58	100.2 ± 10.2	106.2 ± 9.7
Cys478Ala	98.86 ± 8.11	102.5 ± 10.7	105.5 ± 7.0
Ser482Ala	96.85 ± 5.39	98.42 ± 16.22	99.97 ± 8.02
Trp549Ala	95.37 ± 11.19	93.77 ± 6.50	92.81 ± 8.45
Trp549Phe	92.70 ± 3.56	91.84 ± 8.04	90.53 ± 3.80
E-64			
Wild Type	93.09 ± 2.66	104.1 ± 5.6	108.0 ± 6.0
Cys478Ala	98.73 ± 8.81	104.7 ± 8.3	107.4 ± 1.4
Ser482Ala	109.9 ± 13.9	95.98 ± 5.48	105.2 ± 8.4
Trp549Ala	102.0 ± 8.10	98.57 ± 7.27	95.43 ± 8.44
Trp549Phe	105.1 ± 5.20	96.35 ± 4.93	107.1 ± 6.15
Chymostatin			
Wild Type	84.03 ± 7.77	87.43 ± 0.09	90.42 ± 8.16
Cys478Ala	98.57 ± 9.98	67.11 ± 26.39	63.78 ± 30.29
Ser482Ala	123.2 ± 7.1	60.90 ± 17.90	51.57 ± 11.61
Trp549Ala	87.88 ± 2.77	92.60 ± 4.00	105 ± 13.85
Trp549Phe	81.29 ± 3.97	83.72 ± 2.54	80.79 ± 14.51
PMSF			
Wild Type	107.7 ± 4.3	93.07 ± 4.26	71.30 ± 6.67
Cys478Ala	113.89 ± 63.3	70.82 ± 14.22	50.87 ± 28.14
Ser482Ala	102.8 ± 8.3	87.56 ± 28.71	88.37 ± 6.57
Trp549Ala	95.24 ± 4.27	100.32 ± 7.86	91.34 ± 8.85
Trp549Phe	100.12 ± 3.61	83.05 ± 7.79	95.13 ± 4.31

Percent remaining activity of protease compared to protease control with no inhibitor present. Values are means ± S.D. with n ≥ 3.

**Table 3:** The remaining percent activity of enzyme in the presence of metal ions.

nsP2 pro	AGA	AGC	AGG
Nickel			
Wild Type	46.50 ± 4.91	50.14 ± 7.61	53.26 ± 7.80
Cys478Ala	50.77 ± 29.06	65.99 ± 42.26	76.73 ± 38.25
Ser482Ala	59.79 ± 8.07	95.79 ± 7.42	92.02 ± 15.23
Trp549Ala	75.05 ± 8.95	60.20 ± 9.80	37.35 ± 8.09
Trp549Phe	89.49 ± 13.00	67.96 ± 14.34	67.00 ± 5.83
Copper			
Wild Type	82.65 ± 4.02	19.70 ± 8.70	77.41 ± 8.21
Cys478Ala	103.6 ± 65.5	31.49 ± 20.18	87.71 ± 39.68
Ser482Ala	86.42 ± 42.16	34.39 ± 8.24	112.6 ± 40.6
Trp549Ala	83.09 ± 8.99	8.27 ± 2.39	68.68 ± 4.85
Trp549Phe	81.46 ± 12.00	25.93 ± 1.97	60.33 ± 7.26
Cobalt			
Wild Type	200.0 ± 11.2	59.63 ± 0.75	119.3 ± 9.7
Cys478Ala	313.8 ± 52.5	55.63 ± 8.71	114.0 ± 47.2
Ser482Ala	103.1 ± 2.8	52.70 ± 3.06	123.1 ± 22.4
Trp549Ala	161.98 ± 7.12	34.44 ± 11.43	140.67 ± 20.07
Trp549Phe	283.39 ± 44.92	58.52 ± 6.21	150.07 ± 15.84
Magnesium			
Wild Type	83.03 ± 9.53	123.7 ± 44.4	111.2 ± 6.6
Cys478Ala	152.4 ± 49.1	120.9 ± 18.0	121.2 ± 36.1
Ser482Ala	105.6 ± 6.0	111.3 ± 8.7	107.9 ± 7.4
Trp549Ala	97.10 ± 1.61	91.69 ± 5.82	92.07 ± 4.99
Trp549Phe	108.84 ± 5.33	104.89 ± 2.92	93.33 ± 4.81
Zinc			
Wild Type	39.23 ± 26.94	38.76 ± 6.50	39.63 ± 11.55
Cys478Ala	60.38 ± 44.37	44.25 ± 5.36	58.13 ± 50.61
Ser482Ala	nd	21.61 ± 12.89	59.06 ± 11.04
Trp549Ala	71.37 ± 5.84	55.59 ± 4.39	74.17 ± 12.27
Trp549Phe	93.37 ± 2.65	48.88 ± 10.47	71.32 ± 13.34

Percent remaining activity of protease compared to protease control with no metal present. Values are means ± S.D. with n ≥ 3. Nd indicates activity could not be detected, that is, complete inhibition.

**Table 4:** Thermal stability test at 42 °C.

nsP2 pro	AGG
Wild Type	50.36 ± 1.89
Cys478Ala	53.17 ± 8.82
Ser482Ala	50.43 ± 1.71
Trp549Ala	52.86 ± 8.6
Trp549Phe	53.97 ± 4.2

The percent remaining activity of nsP2 pro Wild Type and mutants after incubation at 42 °C for 10 min. The activity of each enzyme incubated at 37 °C was used as a control. Values are means ± S.D. with n ≥ 3.

**Table 5:** Substrate sequences of chikungunya virus nsP2 protease used in this study.

Designated name	Cleavage site (P4–P5′)	Recognition site
AGA	nsP1/2	Nma-**RAGA/GIIETK**(Dnp)-OH
AGC	nsP2/3	Nma-**RAGC/APSYRK**(Dnp)-OH
AGG	nsP3/4	Nma-**RAGG/YIFSSK**(Dnp)-OH

The substrate sequences used in the present study have been previously reported^31^, and this table is adapted from the previous report. Briefly, the three fluorescent substrates designated as AGA, AGC and AGG (as underlined) were synthesized corresponding to the scissile site sequences (shown in upper case bold text) of chikungunya virus non-structural polyprotein (nsP1/2, nsP2/3 and nsP3/4), respectively. A 2-(N-methylamino)benzoyl (Nma) fluorophore group was attached at the amino terminus and a 2,4- dinitrophenyl (Dnp) group attached to the carboxyl terminus of an additional lysine (K) residue.
